# Cognitive fatigue due to exercise under normobaric hypoxia is related to hypoxemia during exercise

**DOI:** 10.1038/s41598-022-14146-5

**Published:** 2022-06-28

**Authors:** Genta Ochi, Ryuta Kuwamizu, Kazuya Suwabe, Takemune Fukuie, Kazuki Hyodo, Hideaki Soya

**Affiliations:** 1 Faculty of Health Sciences, Department of Health and Sports, Niigata University of Health and Welfare, Niigata, 950-3198 Japan; 2 Laboratory of Exercise Biochemistry and Neuroendocrinology, Faculty of Health and Sport Sciences, University of Tsukuba, Ibaraki, 305-8574 Japan; 3 Sports Neuroscience Division, Department of Mind, Advanced Research Initiative for Human High Performance (ARIHHP), Faculty of Health and Sport Sciences, University of Tsukuba, Ibaraki, 305-8574 Japan; 4 Faculty of Health and Sport Sciences, Ryutsu Keizai University, Ibaraki, 301-8555 Japan; 5 Physical Fitness Research Institute, Meiji Yasuda Life Foundation of Health and Welfare, Tokyo, 192-0001 Japan

**Keywords:** Cognitive neuroscience, Human behaviour, Circulation, Neurological disorders

## Abstract

We previously found that a 10-min bout of moderate-intensity exercise (50% maximal oxygen uptake) under normobaric and hypoxic conditions (fraction of inspired oxygen [${{\text{F}}_\text{IO}}_{_{2}}$] = 0.135) reduced executive performance and neural activity in the left dorsolateral prefrontal cortex (DLPFC). To examine whether this cognitive fatigue is due to a decrease in SpO_2_ during exercise, we compared executive performance and related prefrontal activation between two experimental conditions, in which the participants inhaled normobaric hypoxic gas (${{\text{F}}_\text{IO}}_{_{2}}$= 0.135) (hypoxic exercise [HE]) or hypoxic gas adjusted so that SpO_2_ during exercise remained at the resting level (milder hypoxic exercise [ME]). ME condition showed that reaction time in executive performance decreased (*t*[13] = 2.228, *P* < 0.05, *d* = 0.34, paired *t*-test) and left DLPFC activity increased (*t*[13] = -2.376, *P* < 0.05, *d* = 0.63, paired *t*-test) after exercise compared with HE condition. These results showed that the HE-induced reductions in the left DLPFC activity and executive performance were both suppressed in the ME condition, supporting the hypothesis that exercise-induced cognitive fatigue under hypoxic environment is due to hypoxemia during exercise. This may lead to the development of a method of coping with cognitive fatigue due to exercise that causes hypoxemia.

## Introduction

Acute exercise improves executive function in the lateral prefrontal cortex^1–3^. However, exercise may negatively affect not only physical^4–6^, but also executive performance^7^ in hypoxic environments. In fact, by using functional near-infrared spectroscopy (fNIRS), we showed that moderate-intensity exercise in a hypoxic environment (fraction of inspired oxygen [${{\text{F}}_\text{IO}}_{_{2}}$] = 0.135; corresponding to an altitude of 3,500 m) impairs executive function and left dorsolateral prefrontal cortex (l-DLPFC) activity^8^. Executive function is related to motor performance, such as motor coordination^9^ as well as behavioral inhibition and decision-making^10,11^, which are important for physical activity under hypoxic conditions, e.g., mountaineering and high-altitude training. However, the underlying physiological mechanism of executive underperformance (cognitive fatigue) remains unclear.

Cerebral hypoxia, which involves a substantial decrease in percutaneous arterial oxygen saturation (SpO_2_), may be a potential mechanism for cognitive fatigue. SpO_2_ is strongly correlated with oxygen saturation in the prefrontal cortex^12,13^, and exercise-induced low SpO_2_ may indirectly reflect cerebral hypoxia^14^. Indeed, we confirmed that just sitting quietly in severely hypoxic environments (${{\text{F}}_\text{IO}}_{_{2}}$ = 0.115; equivalent to an altitude of 5000 m), where SpO_2_ decreases below 80%, reduces executive function^15^. In addition, previous studies, wherein SpO_2_ reduced to 80% during moderate-intensity exercise on the semi-recumbent cycle ergometer, even in moderately hypoxic environments (${{\text{F}}_\text{IO}}_{_{2}}$ = 0.125–0.135; equivalent to an altitude of 3500–4100 m), reported impaired executive function^7,8^. These results suggest that severely low SpO_2_ caused by exercise in a hypoxic environment might affect cortical activation, which leads to cognitive fatigue. Interestingly, inhibition of motor cortex activation and cerebral hypoxia is thought to be a factor in central fatigue, which limits exercise execution^16,17^. Moreover, 2 weeks of acclimatization to hypoxic environments suppressed cerebral hypoxia by suppressing low SpO_2_ during exercise under hypoxic conditions and ameliorated the reduction in the central command from the motor cortex (central fatigue)^18^. If cerebral hypoxia associated with low SpO_2_ is also a factor in cognitive fatigue, then cognitive fatigue can be improved by suppressing low SpO_2_ during exercise in hypoxic environments. However, while previous studies on the effects of cerebral hypoxia associated with low SpO_2_ during exercise have focused on the motor cortex, no studies have focused on cognitive fatigue associated with decreased prefrontal neural activity.

Using fNIRS, we previously found that a 10-min bout of moderate-intensity exercise (50% maximal oxygen uptake: ${\dot{\text{V}}\text{O}}_{{2{\text{peak}}}}$ ) under normobaric and hypoxic conditions (${{\text{F}}_\text{IO}}_{_{2}}$ = 0.135) reduced executive performance measured by the color-word Stroop task (CWST) and neural activity in the l-DLPFC, which is responsible for executive functions^8^. One of the potential physiological reasons for this would be the decrease in SpO_2_ that occurs during exercise in hypoxic environments^8^. To test this hypothesis, we aimed to examine whether low SpO_2_ is due to executive underperformance after exercise in hypoxic environments. We compared moderate-intensity exercise in a moderately hypoxic environment (${{\text{F}}_\text{IO}}_{_{2}}$ = 0.135), which impairs executive function, with exercise in conditions in which the participant breathed a mildly hypoxic gas (${{\text{F}}_\text{IO}}_{_{2}}$= 0.161 ± 0.018) during exercise so that their SpO_2_ did not decrease from the resting state.

## Results

### Physiological parameters

**Figure 1:**
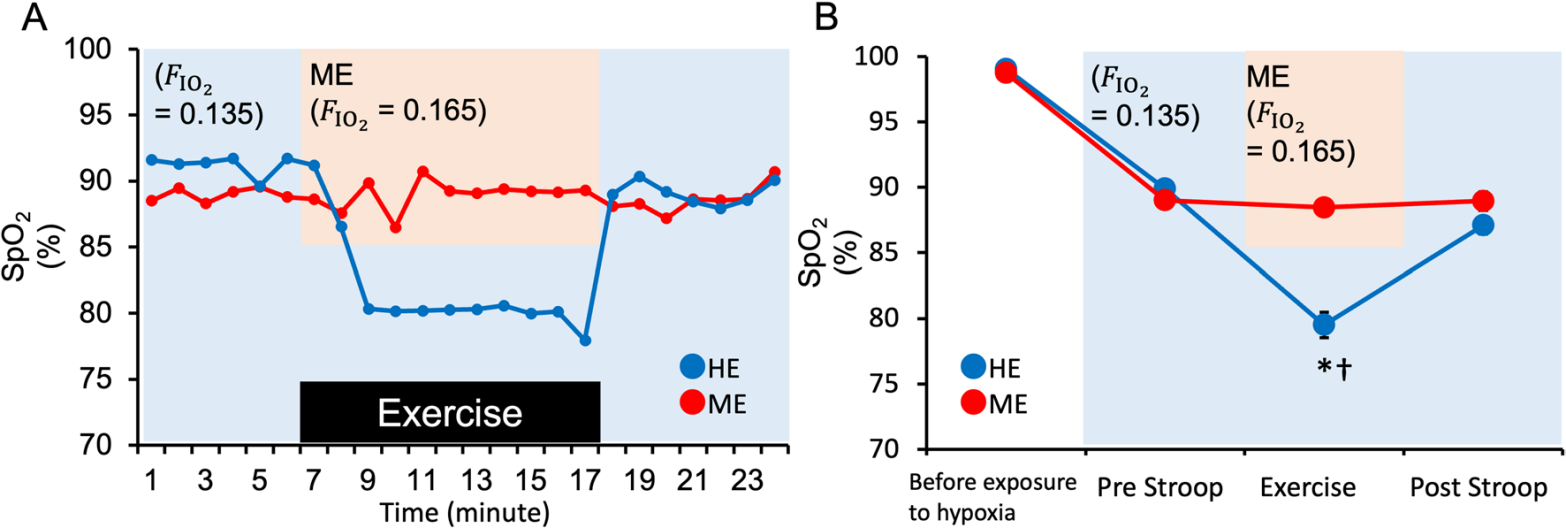
(**A**) A typical example of the percutaneous arterial oxygen saturation (SpO_2_) during exercise. Immediately before exercise, the inhaled hypoxic gas was switched from hypoxia (${{\text{F}}_\text{IO}}_{_{2}}$= 0.135; blue background) to a milder hypoxic gas (${{\text{F}}_\text{IO}}_{_{2}}$= 0.161 ± 0.018; orange background), which was adjusted so that SpO_2_ during exercise remained at the resting level in the ME condition (red). Immediately after the end of the exercise, the inhaled hypoxic gas was again returned to hypoxia (${{\text{F}}_\text{IO}}_{_{2}}$ = 0.135). (**B**) The mean and standard deviation of SpO_2_ under hypoxic exercise (HE) (blue) and milder hypoxic exercise (ME) (red) conditions. Values are presented as mean ± standard error. *P < 0.05 versus (vs.) ME condition, †*P* < 0.05 vs. pre-Stroop.

Table 1 summarizes the results of the physiological parameters. Heart rate (HR), ratings of perceived exertion (RPE), ventilation (${\dot{{V}}}_\text{E}$), end-tidal carbon dioxide concentration (ETCO_2_), and SpO_2_ were subjected to repeated measures two-way analysis of variance (ANOVA) with condition (hypoxic exercise [HE]/milder hypoxic exercise [ME]) and time (before exposure to hypoxia/pre-Stroop/during exercise/post-Stroop) as within-subject factors. Other respiratory gas parameters are shown in the Supplementary materials. There was a significant interaction between time and condition for HR (*F*[3, 39] = 10.52, *P* < 0.001, *η*^*2*^_*p*_ = 0.44) and ETCO_2_ (*F*[3, 39] = 22.626, *P* < 0.001, *η*^*2*^_*p*_ = 0.64). We found significant increases in HR and ETCO_2_ during exercise compared to during pre-Stroop sessions in both conditions. HR during exercise was higher during HE than during ME, and ETCO_2_ during exercise was lower during HE than during ME. There were the significant main effects in RPE and ${\dot{{V}}}_\text{E}$, and there was a predominant increase during exercise under ME and HE conditions. There was a significant interaction between time and condition for SpO_2_ (*F*[3, 39] = 83.995, *P* < 0.001, *η*^*2*^_*p*_ = 0.87). We also found a significant decrease in SpO_2_ during exercise compared to during the pre-Stroop session in the HE condition. Post hoc analyses showed that SpO_2_ values during exercise periods under the HE condition were lower than those under the ME condition (Fig. 1). In the HE condition, SpO_2_ during exercise was comparable to that in our previous study (this study, 79.5 ± 3.6%; previous study, 81.7 ± 1.4%)^8^; we confirmed that the experimental model in which cognitive fatigue occurs was replicated in this study.

**Table 1:** Physiological parameters.

Variable	Condition	Before exposure to hypoxia	Pre-Stroop	During exercise	Post-Stroop
HR (bpm)	ME	69.6 (1.8)	76.2 (2.2)	128.7 (3.1)^†^	85.7 (2.5)^†^
RPE (point)			7.9 (0.7)	12.9 (0.5)^†^	
${\dot{{V}}}_\text{E} \left( {{\text{L}}/\min } \right)$		10.8 (0.5)	10.3 (0.4)	42.8 (2.2)^†^	10.4 (0.5)
ETCO_2_ (%/min)		5.1 (0.1)	5.3 (0.0)	6.1 (0.1)^†^	5.2 (0.0)
HR (bpm)	HE	70.3 (2.6)	76.7 (2.8)	138.7 (2.8)*^†^	89.2 (3.1)*^†^
RPE (point)			7.8 (0.5)	13.9 (0.5)^†^	
${\dot{{V}}}_\text{E} \left( {{\text{L}}/\min } \right)$		10.2 (0.9)	9.3 (0.7)	44.6 (3.2)^†^	9.3 (0.7)
ETCO_2_ (%/min)		5.1 (0.1)	5.3 (0.1)	5.7 (0.1)*^†^	5.2 (0.1)

The value of each indicator is averaged over the 3 min before exposure to hypoxia, during the Stroop task (Pre and Post, 6.5 min each), and during exercise (10 min). HE, hypoxic exercise; ME, milder hypoxic exercise; HR, heart rate; RPE, ratings of perceived exertion; ${\dot{{V}}}_\text{E}$, ventilation; ETCO_2_, end-tidal carbon dioxide concentration; bpm, beats per minute.

Values are presented as mean (standard error). *P < 0.05 versus (vs.) ME condition, ^†^*P* < 0.05 vs. pre-Stroop.

### Executive performance: Stroop interference

**Figure 2:**
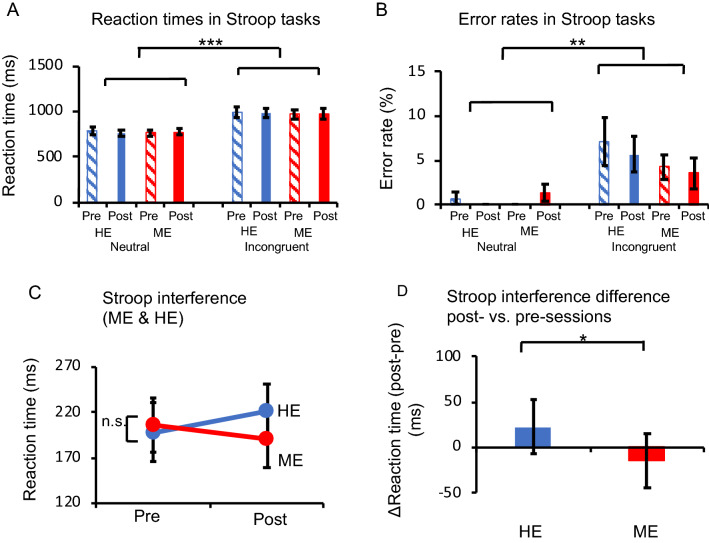
(**A**) Comparison of the reaction time (RT) between the incongruent and neutral conditions. The incongruent condition exhibits a significantly slower RT than the neutral condition (***P < 0.001). (**B**) Comparison of the error rate between the incongruent and neutral conditions. Significant Stroop interference effects are observed (**P < 0.005). (**C**) The mean difference of the RT in incongruent and neutral trials indicates Stroop interference for each condition. (**D**) Stroop interference (RT) differences between post- and pre-sessions for each condition. Stroop interference differences are significantly more positive in the hypoxic exercise (HE) condition than in the milder hypoxic exercise (ME) condition (*P < 0.05). Error bars indicate the standard error.

We first examined whether a general tendency in the CWST could be reproduced under all conditions. The reaction time (RT) and error rate were subjected to a repeated-measures three-way ANOVA with trial (incongruent/neutral), condition (HE/ME), and session (pre/post) as within-subject factors. ANOVA revealed significant main effects of trial on the RT (*F*[1, 13] = 60.274, *P* < 0.001, *η*^*2*^_*p*_ = 0.82, Fig. 2A) and error rate (*F*[1, 13] = 12.138, *P* < 0.005, *η*^*2*^_*p*_ = 0.48, Fig. 2B). These results verified that Stroop interference was generally observed in all the sessions used in this experiment as in our previous studies^1–3,8,15,38–40,42,45^. Therefore, to clarify the effect of an acute bout of exercise on a specifically defined cognitive process, we focused on analyses of Stroop interference (incongruent – neutral). ANOVA for the RT of Stroop interference revealed a significant interaction between condition and session factors (*F*[1, 13] = 4.964, *P* < 0.05, *η*^*2*^_*p*_ = 0.28, Fig. 2C). There was no significant interaction or main effect on the error rate.

Next, to examine the interaction for the RT, we calculated the difference in the degree of Stroop interference between post- and pre-sessions: ([incongruent–neutral] of pre-session–[incongruent–neutral] of post-session); contrasts for both the HE and ME conditions were calculated, and the differences between them were compared. The RT difference was significantly more negative in the HE condition than in the ME condition (*t*[13] = 2.228, *P* < 0.05, *d* = 0.34, paired *t*-test; Fig. 2D). These results demonstrate that the negative effect of exercise on executive performance under the HE condition was diminished under the ME condition.

### Results of fNIRS

**Figure 3:**
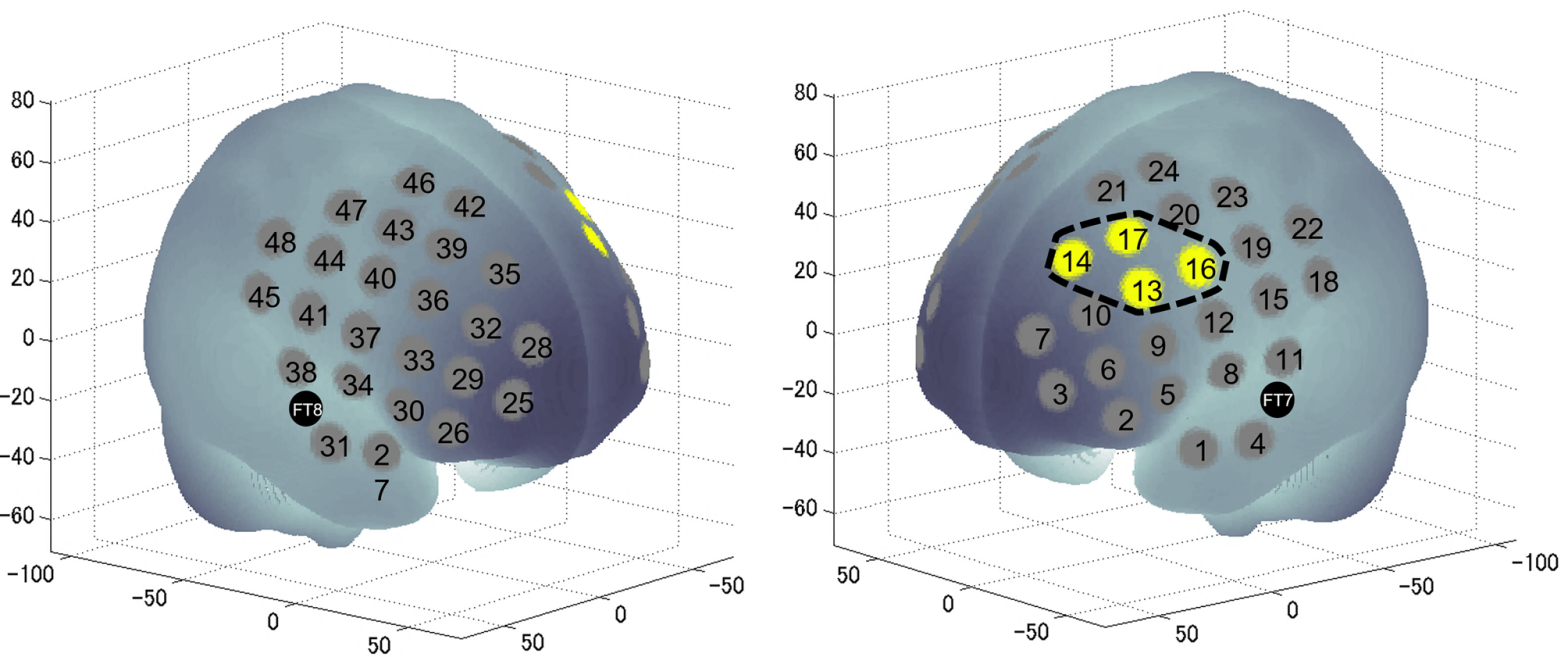
The spatial profiles of functional near-infrared spectroscopy channels and region of interest segmentation used in the current study; they were introduced in previous studies^46,50^. Channel numbers and FT7 and FT8 in the international 10–20 electroencephalography standard positions are denoted above the corresponding locations. The channels enclosed by the black broken lines were defined as the l-DLPFC, and their data were integrated for further analyses.

**Figure 4:**
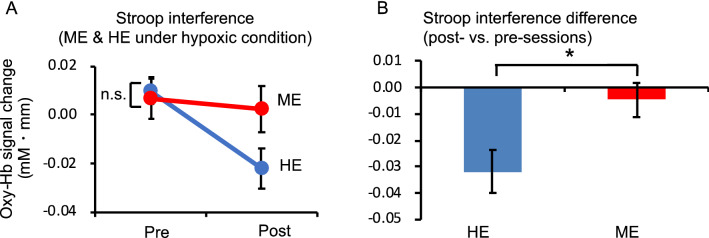
(**A**) Stroop interference differences between post- and pre-sessions for oxygenated hemoglobin (oxy-Hb) signal contrasts in both conditions. (**B**) Oxy-Hb signal differences for the hypoxic exercise (HE) condition are significantly lower than those for the milder hypoxic exercise (ME) condition (*P* < 0.05). Error bars indicate the standard error.

Since a previous study^8^ showed that the increased RT of Stroop interference by moderate exercise under hypoxic conditions was associated with decreased l-DLPFC activity, we assessed the effects of moderate exercise under hypoxic conditions on l-DLPFC activation, focusing on Stroop interference (Fig. 3). The oxygenated hemoglobin (oxy-Hb) signal differences (incongruent–neutral) in response to Stroop interference in the l-DLPFC were analyzed with repeated measures two-way ANOVA, including condition (HE/ME) and session (pre/post) as within-subject factors. In this design, the effect of an acute bout of moderate exercise on Stroop interference was expected to appear as an interaction between the two factors, because pre-HE and pre-ME were virtually identical. ANOVA performed on the regions of interest (ROIs) of l-DLPFC revealed a significant interaction (*F*[1, 13] = 8.63, *P* < 0.05, *η*^*2*^_*p*_ = 0.40, Fig. 4A). There were no significant differences between pre-sessions. To clarify the exercise-session interaction in the ROIs of l-DLPFC, differences in hemodynamic responses due to Stroop interference between the post- and pre-sessions were analyzed. Oxy-Hb signal differences in response to Stroop interference in the l-DLPFC were significantly lower in the HE condition than in the ME condition (*t*[13] = −2.376, *P* < 0.05, *d* = 0.63, paired *t*-test; Fig. 4B). However, no significant interactions or main effects of deoxygenated hemoglobin (deoxy-Hb) were observed.

### Association of executive performance and l-DLPFC activity with physiological parameters

We examined the correlation between the delayed Stroop interference-related RT and altered ${\dot{{V}}}_\text{E}$, ETCO_2_, and SpO_2_ during exercise in the HE condition. There was no notable correlation between the RT and any parameter (${\dot{{V}}}_\text{E}$ : *r* = 0.203, *P* > 0.05; ETCO_2_: *r* = − 0.109, *P* > 0.05; SpO_2_: *r* = 0.08, *P* > 0.05). Moreover, we examined the correlation between reduced activation in the l-DLPFC and altered ${\dot{{V}}}_\text{E}$ , ETCO_2_, and SpO_2_ during exercise under the HE condition. There was no significant correlation between l-DLPFC activation and any of the parameters (${\dot{{V}}}_\text{E}$: *r* = -0.406, *P* > 0.05; ETCO_2_: *r* = − 0.025, *P* > 0.05; SpO_2_: *r* = 0.227, *P* > 0.05).

## Discussion

This study aimed to examine the involvement of low SpO_2_ during exercise in executive underperformance and prefrontal deactivation caused by exercise in hypoxic environments. We found that suppression of low SpO_2_ during exercise in hypoxic environments diminished the negative effects on CWST performance and task-related l-DLPFC activity. These result suggest that exercise-induced executive underperformance under hypoxic environment is due to decrease in SpO_2_ during exercise.

Behavioral measurements revealed a shorter RT and lower error rate in neutral trials than in incongruent trials. Therefore, we confirmed that Stroop interference could be stably induced before and after an acute bout of moderate exercise under both conditions. Based on these data, we first compared the effects of the HE and ME conditions on Stroop interference. Stroop interference increased after 10 min of exercise in the HE condition compared with the ME condition. This finding is consistent with those of previous studies that have demonstrated that an acute bout of moderate exercise under normobaric hypoxic conditions decreases inhibitory control function^8^. However, there was no delay in Stroop interference in the ME condition. The aspirated oxygen concentration and SpO_2_ during the CWST did not differ between the two conditions, suggesting that the decline in executive function was associated with hypoxemia during exercise.

In the subsequent analysis, we examined the brain regions that were deactivated after exercise in a hypoxic environment. Previous studies have confirmed that the activity of the l-DLPFC decreases after exercise under normobaric hypoxic conditions^8^. Therefore, in the present study, we also focused on the decreased activity of the l-DLPFC in response to increased Stroop interference. As a result, a decrease in neural activity of the l-DLPFC was observed after exercise in the HE condition, similar to the results of previous studies, whereas in the ME condition, the decrease in neural activation after exercise improved. These results suggest that the suppression of low behavioral data after exercise in the ME condition may involve the suppression of task-specific decreased activity in the l-DLPFC.

In this study, SpO_2_, ${\dot{{V}}}_\text{E}$, and ETCO_2_, which are involved in cerebral vasodilation during the CWST, were not significantly different before and after exercise or between the conditions. This finding suggests that the negative effect on CWST performance and l-DLPFC activity is related to altered physiological parameters during exercise. Our previous study showed that CWST performance was impaired in severely hypoxic environments where SpO_2_ decreased below 80%^15^, suggesting that the impaired executive function in this study was due to low SpO_2_ during exercise. Similarly, a previous study reported that hypoxemia during exercise in hypoxic environments is associated with decreased neural activity in the motor cortex and decreased motor performance^18^. In addition, moderate exercise under normobaric hypoxic conditions (${{\text{F}}_\text{IO}}_{_{2}}$ = 0.10) led to a decreased cerebral metabolic rate for oxygen (CMRO_2_)^19^, and a reduction in CMRO_2_ with exposure to normobaric hypoxic conditions (${{\text{F}}_\text{IO}}_{_{2}}$ = 0.08) did not return to baseline after re-oxygenation^20^. Therefore, we considered that exercise-induced cognitive decline in the HE condition might be due to the low SpO_2_ during exercise.

During exercise in a hypoxic environment, SpO_2_ decreases due to the ventilation-to-perfusion ratio inequality and diffusion limitation which is due to the reduction in the erythrocyte transit time in the pulmonary capillaries with the increase in cardiac output^21^. Although a decrease in SpO_2_ limits oxygen supply to the brain, only low SpO_2_ but with normal cerebral blood flow might not affect the neural activity of an intact organism^22^. This is thought to be due to the cerebral blood flow increase as the SpO_2_ decreases^23–25^. However, this vascular response is not uniform across the brain. It has been reported that changes are greater in paleocortical regions than in neocortical regions, suggesting that the blood flow change during decreasing SpO_2_ may protect regions of the brain with essential homeostatic roles^26^. In addition, since exercise in a hypoxic environment has been suggested to cause competition for blood flow distribution between muscles and the brain^27^, it is possible that this cerebral blood flow response in the prefrontal cortex was more inadequate, resulting in impaired executive function. Although the mechanism by which neural activity decreases under cerebral hypoxia still remains unclear, one possible cause is neural deactivation associated with inflammatory response. In an animal study, it has been shown that hypoxia is accompanied by an inflammatory response via hypoxia-inducible factor and nuclear factor-kB^28^, and the combination of hypoxia and inflammation rapidly decreases the neural excitability in the rodent hippocampal CA1 neurons^29^. An acute exercise, on the other hand, also induces acute inflammatory markers, which results in increased inflammatory cytokine levels in the animal cortex^30^. If so, in this study, it is possible that the combined effects of hypoxia and inflammation occur in the human prefrontal cortex with hypoxic exercise, which in turn may cause cognitive fatigue. This hypothesis, however, needs to be verified in the future. A limitation of this study is that we were unable to evaluate the relationship between oxygen metabolism in the brain during exercise and post-exercise decline in executive function. Since SpO_2_ is strongly correlated with prefrontal oxygen saturation^12,13,31^, it is assumed that cerebral hypoxia during exercise in the HE condition decreases executive function and l-DLPFC activity. However, in the present study, no significant correlation was found between decreased SpO_2_ and decreased executive function during exercise, and it remains unclear how low SpO_2_ is related to negative effects on executive function. Although fMRI and fNIRS still have issues in evaluating neural activity during exercise, such as the effects of body movement and skin blood flow, transcranial magnetic stimulation (TMS) has been used to evaluate neural activity in the motor cortex during exercise under hypoxia. Future evaluation of prefrontal neural activity and oxygen metabolism during exercise under hypoxia using TMS will help elucidate the mechanism of cognitive fatigue.

Another limitation of the present study is that it was conducted in healthy adults who do not normally engage in strenuous exercise, so there was insufficient inter-subject evaluation. In fact, elite mountaineers, compared to non-mountaineers, showed a smaller decrease in SpO_2_ in the quadriceps muscle under hypoxic conditions (${{\text{F}}_\text{IO}}_{_{2}}$ = 0.125) and might maintain oxygen supply to the tissues^32^. In addition, no sex-related differences were detected for reaction time and DLPFC activity or physiological indices. This lack of difference is supposedly due to the small sample size of female participants. The ventilatory response in hypoxic environments in women is higher than that in men^33^; some authors hypothesize that women have a strong protective mechanism against acute exposure to hypoxia^34^. It is possible that cognitive fatigue due to exercise under hypoxic conditions is also attenuated in women, but this needs to be verified in the future. Furthermore, some previous studies have reported that acclimatization to hypoxic environments increased the oxygen supply to the brain during the resting state^35^ and exercise^18^ in hypoxic environments compared to before acclimatization and improved hypoxia-induced motor cortex hypoactivity^18^. Based on the present study, further studies are needed to examine whether acclimatization to hypoxic environments improves cognitive fatigue in mountaineers and trail runners who have been acclimatized to hypoxic environments through hypoxic training. This may lead to the elucidation of the actual situation of exercise-induced cognitive fatigue and the development of methods to cope with it.

In conclusion, the current study revealed that the decline in executive function and l-DLPFC activity after moderate-intensity exercise under hypoxic environments could be prevented by suppressing the decrease in SpO_2_ during exercise, suggesting that the exercise-induced cognitive fatigue under hypoxic environment is due to hypoxemia during exercise. The results of this research will contribute to the elucidation of the mechanism of cognitive fatigue caused by a decrease in blood oxygen concentration due to exercise, as well as activities in hypoxic environments, e.g., high altitude, and to the development of methods to cope with such central fatigue.

## Methods

### Ethics statements

This study was approved by the Institutional Ethics Committee of the University of Tsukuba, Faculty of Health and Sport Sciences (approval number: Tai 025–120) and was conducted in accordance with the latest version of the Declaration of Helsinki. The study participants provided written informed consent for participation and publication of their details.

### Participants

Fourteen right-handed young adults (12 men and 2 women) participated in this study (Table 2). The sample size was determined by assuming that the effects of exercise in hypoxic environments would be similar to those in our previous study^8^. All participants were Japanese native speakers, healthy, and naive to the experimental procedures for which they volunteered. None of the participants had a history of neurological, psychiatric, or respiratory disorders or a disease requiring medical care. All the participants had normal or corrected-to-normal vision and normal color vision. All participants were asked to refrain from exercise and the consumption of alcohol and caffeine for at least 24 h prior to each experiment to control for external factors that could affect cardiovascular and executive functions. Post-hoc sensitivity analysis performed based on this sample with 80% power and 0.05 alpha demonstrated sufficient sensitivity to detect repeated-measures effects exceeding *f* = 0.40 and *t*-test differences exceeding *d* = 0.81 (with a two-tailed alpha), as computed using G*Power (3.1.9.2).

**Table 2:** Participants’ characteristics.

	Age (years)	Height (cm)	Weight (kg)	${\dot{\text{V}}\text{O}}_{{2{\text{peak}}}}$(mL·kg·min^-1^)	Workload (W)
Average (SD)	21.4 (1.7)	171.7 (7.1)	63.3 (7.2)	44.7 (9.5)	116.6 (23.7)

Age, height, weight, peak oxygen intake (${\dot{\text{V}}\text{O}}_{{2{\text{peak}}}}$), and relative workload for moderate-intensity exercise are presented as the mean and standard deviation for the 14 participants.

### Experimental procedures

**Figure 5:**
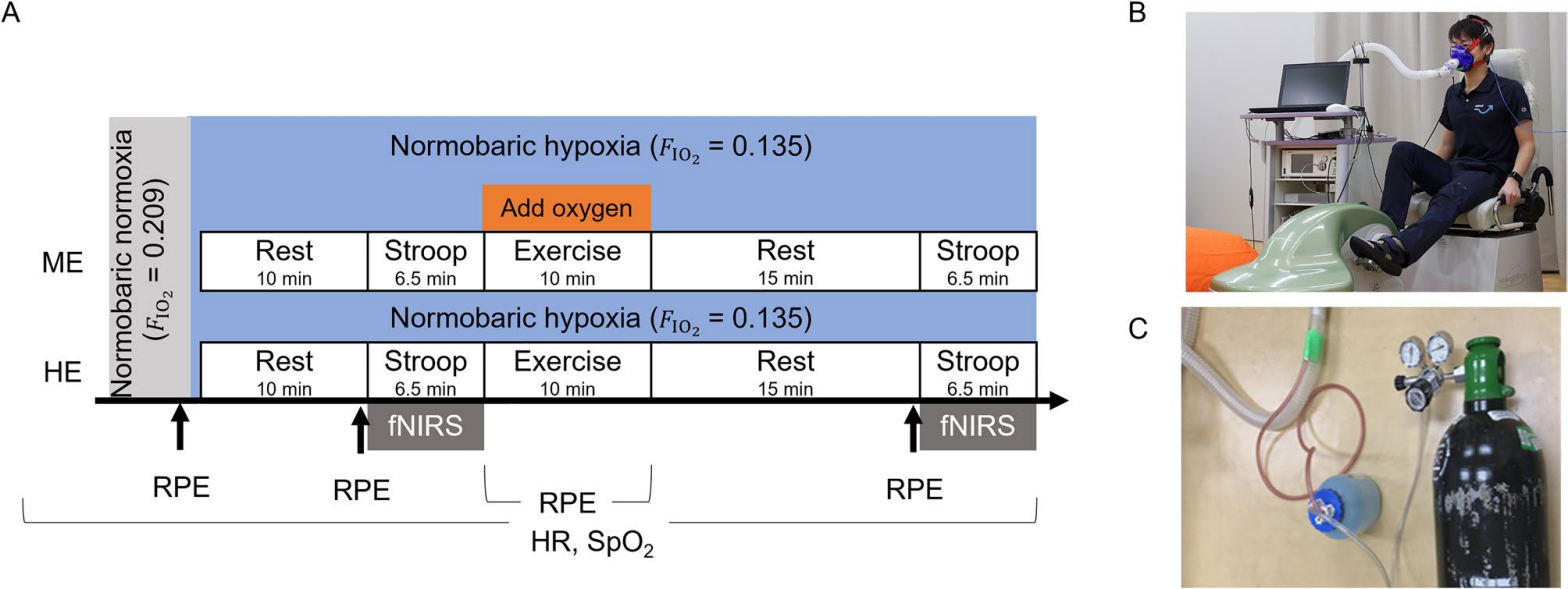
(**A**) The two conditions, breathing a moderately hypoxic gas (hypoxic exercise [HE]) and breathing a milder hypoxic gas, during which oxygen was added to the moderately hypoxic gas to maintain the oxygen saturation (SpO_2_) level during milder hypoxic exercise (ME). Cortical hemodynamic changes were monitored with functional near-infrared spectroscopy (fNIRS) while participants performed the Stroop task. HR, heart rate. (**B**) In both conditions, the exercise and color-word Stroop task (CWST) were performed on a recumbent cycle ergometer. Hypoxic gas (${{\text{F}}_\text{IO}}_{_{2}}$ = 0.135) stored in a Douglas bag was inhaled through a mask. (**C**) In the ME condition, SpO_2_ was adjusted during exercise by adding oxygen gas with humidity to prevent the participant’s throat from drying out to the hose connecting the Douglas bag to the mask.

On the first day, participants underwent a graded exercise test to measure their ${\dot{\text{V}}\text{O}}_{{2{\text{peak}}}}$ and determine the appropriate individual intensity for moderate exercise, which was defined as 50% of a participant’s ${\dot{\text{V}}\text{O}}_{{2{\text{peak}}}}$ based on the American College of Sports Medicine's classification of physical activity intensity^36^. The detailed procedures for the graded exercise test were the same as those in our previous study^8^. The participants practiced the CWST twice before being subjected to the main experimental conditions. A few days after the first visit, two main experimental conditions were conducted in a single-blind (participant being blinded) crossover study design: exercise under moderate normobaric hypoxia (HE) or ME, which improved the inhaled oxygen concentration to suppress SpO_2_ during exercise. All participants participated in both the HE and ME conditions, each on separate days, with the order counterbalanced across participants (Fig. 5). In both conditions, participants underwent the CWST before and 15 min after 10 min of moderate-intensity exercise on a recumbent cycle ergometer (Strength Ergo 240 W, Mitsubishi Electric Corp., Tokyo, Japan) at 60 revolutions per minute, based on our previous study methods^8^. Cortical hemodynamic changes in the l-DLPFC were monitored using fNIRS while the participants performed the CWST.

In the HE condition, participants breathed hypoxic gas, as in our previous study^8^, which was a mixture of 13.5% oxygen and 0.03% carbon dioxide in nitrogen (${{\text{F}}_\text{IO}}_{_{2}}$ = 0.135; equivalent to an altitude of approximately 3,500 m) through a mask connected to a Douglas bag. In the ME condition, as in the HE condition, participants breathed a moderately hypoxic gas during the CWST, but only during exercise; they breathed a milder hypoxic gas (${{\text{F}}_\text{IO}}_{_{2}}$ = 0.161 ± 0.018) to regulate the hypoxic gas so their SpO_2_ did not decrease from the resting state. To adjust the oxygen concentration, oxygen was injected directly into the hose while monitoring the SpO_2_ during exercise. Expired air was exhausted directly outside the mask so participants did not re-breathe it. The participants were exposed to a moderately hypoxic gas 10 min before the pre-Stroop session while sitting on a cycle ergometer. HR was monitored by a heart rate monitor (V800, Polar Electro, Kempele, Finland), SpO_2_ was monitored by a pulse oximeter (OLV-3100, Nihon Kohden, Tokyo, Japan) placed on the left earlobe, and exhaled gas was monitored every minute by a gas analyzer (Aeromonitor AE-310S; Minato Medical Science, Osaka, Japan). The participants’ RPE^37^ were recorded before exposure to hypoxia, every minute during exercise, and before the CWST.

### Behavioral measurements

The CWST^1–3,8,15,38–42^ was adopted in an event-related design. The CWST, which included two rows containing letters or words, was presented on a screen, and the participants were instructed to decide whether the color of the letters or words in the top row corresponded to the color name presented in the bottom row. Participants pressed a “yes” or “no” button with their right forefinger or middle finger to respond. The RT and error rates were measured.

The CWST consisted of three trials, including 10 neutral, 10 congruent, and 10 incongruent trials. For neutral trials, the top row contained sets of X’s (XXXX) written in red, blue, green, or yellow, and the bottom row contained the words “RED,” “BLUE,” “GREEN,” or “YELLOW” written in black. For congruent trials, the top row contained the words “RED,” “BLUE,” “GREEN,” or “YELLOW” written in a color congruent with that of the bottom row. For incongruent trials, the color word in the top row was written in an incongruent color to produce interference between the color of the word and the color name. All words were written in Japanese. The correct answer rate assigned to “yes” and “no” was 50%. Each stimulus was separated by an inter-stimulus interval showing a fixation cross for 9–13 s to avoid predicting the timing of the subsequent trial^1,2,8,15,38–40,42^. The stimulus remained on the screen until a response was given or for 2 s. In the present study, Stroop interference, a specifically defined cognitive process, was used to elucidate the effect of an acute bout of moderate exercise under hypoxic conditions on executive function. Therefore, the (incongruent-neutral) contrast, which is assumed to represent Stroop interference, was calculated.

### fNIRS measurements

We used a multichannel fNIRS optical topography system (ETG-7000, Hitachi Medical Corporation, Chiba, Japan) set with two wavelengths of near-infrared light (785 and 830 nm). We analyzed the optical data from fNIRS based on the modified Beer–Lambert law^43^, as previously described^44^. This method allowed us to calculate signals reflecting the oxy-Hb, deoxy-Hb, and total hemoglobin concentration changes, calculated in millimolar-millimeters (mM⋅mm)^44^. The composition of the fNIRS probe holder and its placement followed the same procedure used in our previous studies^1–3,8,38–40,42,45^. To register fNIRS data in the Montreal Neurological Institute (MNI) space, we used virtual registration^46,47^. Briefly, this method enables the placement of a virtual probe holder on the scalp by stimulating the holder’s deformation and registering probes and channels onto a reference brain in a preconstructed magnetic resonance imaging database^48,49^. We probabilistically estimated the MNI coordinate values for the fNIRS channels to obtain the most likely estimate of the location of the given channels for the group of participants and the spatial variability associated with the estimation^50,51^.

### Analysis of the fNIRS data

In this study, neural activity related to the CWST was evaluated by examining changes in oxy-Hb, as shown in our previous studies^1–3,8,38–40,42,45^. Individual timeline data for the oxy-Hb signal of each channel were preprocessed with a band-pass filter using a cut-off frequency of 0.04 Hz to remove baseline drift and 0.3 Hz to filter out heartbeat pulsations. Channel-wise and subject-wise contrasts were obtained by calculating the inter-trial mean of differences between the oxy-Hb signals of the peak (6–10 s after trial onset) and baseline (0–2 s before trial onset) periods based on our study^8^. The contrasts were calculated as prefrontal activation elicited by a cognitive task, and the contrasts obtained were subjected to second-level random-effects group analysis.

Based on a method widely used in anatomical labeling systems, such as the LBPA40^49^, channels 13, 14, 16, and 17 were combined to analyze l-DLPFC activity, which was found to decrease after moderate-intensity exercise under hypoxic conditions in a previous study^8^.

### Statistical analysis

The HR, RPE, and SpO_2_ were subjected to repeated measures two-way ANOVA with condition (HE/ME) and time (before exposure to hypoxia/pre-Stroop/during exercise/post-Stroop) as within-subject factors. The RT and error rate were subjected to repeated measures three-way ANOVA with trial (incongruent/neutral), condition (HE/ME), and session (pre/post) as within-subject factors to examine whether the general tendencies for the Stroop task could be reproduced in all conditions. The Stroop effect associated with acute moderate exercise on all outcome measures was analyzed using repeated measures two-way ANOVA with condition (HE/ME) and session (pre/post) as within-subject factors. When a significant *F*-value was obtained, a post hoc test using the Bonferroni method for multiple corrections was applied to identify significant differences among the mean values.

Moreover, to clarify the relationships of physiological parameters (SpO_2_, ETCO_2_, and ${\dot{{V}}}_\text{E}$) during exercise with executive performance and task-related brain activation, we conducted parametric Pearson correlation analyses.

All data are presented as mean ± standard error. Statistical significance was set a priori at *P* < 0.05 for all comparisons. Statistical analyses were performed using the Statistical Package for the Social Sciences (SPSS) version 26 (SPSS Inc., Chicago, IL, USA).

## Supplementary Information

Supplementary Information.

## Data Availability

The datasets generated and/or analyzed during the current study are available from the corresponding author on reasonable request.
