# Supplementary material for: Cognitive fatigue due to exercise under normobaric hypoxia is related to hypoxemia during exercise
Source: Sci Rep. 2022 Jun 28;12:9835. doi: 10.1038/s41598-022-14146-5 (PMC9240057; doi:10.1038/s41598-022-14146-5)
Supplement: Supplementary file 1 — Supplementary Information. [file 41598_2022_14146_MOESM1_ESM.docx]

**Supplementary data**

Cognitive fatigue due to exercise under normobaric hypoxia is related to hypoxemia during exercise

**Genta Ochi^1,2,3^, Ryuta Kuwamizu^2^, Kazuya Suwabe^2,3,4^, Takemune Fukuie^2^, Kazuki Hyodo^5^, Hideaki Soya^2,3*^**

^1^Faculty of Health Sciences, Department of Health and Sports, Niigata University of Health and Welfare, Niigata 950-3198, Japan

^2^Laboratory of Exercise Biochemistry and Neuroendocrinology, Faculty of Health and Sport Sciences, University of Tsukuba, Ibaraki 305-8574, Japan

^3^Department of Sports Neuroscience, Advanced Research Initiative for Human High Performance (ARIHHP), Faculty of Health and Sport Sciences, University of Tsukuba, Ibaraki 305-8574, Japan

^4^Faculty of Health and Sport Sciences, Ryutsu Keizai University, Ibaraki 301-8555, Japan

^5^Physical Fitness Research Institute, Meiji Yasuda Life Foundation of Health and Welfare, Tokyo 192-0001, Japan

**Table S1.** Changes in respiratory gas parameter

| Variable | Condition | Before exposure to hypoxia | Pre-Stroop | During exercise | Post-Stroop |
| --- | --- | --- | --- | --- | --- |
| $\dot{V}_{O_{2}}$ (ml/min) | ME | 262.1 (49.9) | 259.4 (48.9) | 1479.9 (272.4)^†^ | 260.9 (73.6) |
| $\dot{V}_{{CO}_{2}}$ (ml/min) |  | 257.9 (49.7) | 232.7 (47.6) | 1417.1 (284.2)^†^ | 231.3 (54.1) |
| RER |  | 0.89 (0.03) | 0.88 (0.03) | 0.96 (0.06)^†^ | 0.85 (0.05) ^†^ |
| ETO_2_ (%/min) |  | 12.6 (0.7) | 7.8 (0.3) | 9.3 (0.7)^†^ | 7.9 (0.7) |
| $\dot{V}_{O_{2}}$ (ml/min) | HE | 259.4 (61.1) | 258.8 (39.9) | 1523.8 (439.3)^†^ | 278.1 (40.0) |
| $\dot{V}_{{CO}_{2}}$ (ml/min) |  | 259.4 (61.1) | 228.3 (31.3) | 1486.2 (263.2)*^†^ | 226.3 (23.7) |
| RER |  | 0.90 (0.05) | 0.90 (0.07) | 1.00 (0.05)^†^ | 0.85 (0.14) ^†^ |
| ETO_2_ (%/min) |  | 12.7 (0.9) | 7.8 (0.4) | 7.8 (0.3)^*^ | 7.5 (0.4) ^†^ |

$\dot{V}_{O_{2}}$, volume of oxygen uptake; $\dot{V}_{{CO}_{2}}$, volume of oxygen output; RER, respiratory exchange ratio; ETO_2_, end-tidal oxygen concentration.

Values are presented as mean (standard error). ^*^*P* < 0.05 versus (vs.) ME condition, ^†^*P* < 0.05 vs. pre-Stroop

There was a significant main effect of time for $\dot{V}_{O_{2}}$ (*F*[3, 39] = 219.999, *P* < 0.001, *η^2^_p_* = 0.94) and RER (*F*[3, 39] = 27.182, *P* < 0.001, *η^2^_p_* = 0.68). There were significant increases in $\dot{V}_{O_{2}}$and RER during exercise compared to during pre-Stroop sessions in both conditions. RER significantly decreased during post-Stroop sessions compared to during pre-Stroop sessions in both conditions. There were significant interaction between time and condition for $\dot{V}_{{CO}_{2}}$ (*F*[3, 39] = 11.96, *P* < 0.001, *η^2^_p_* = 0.48) and ETO_2_ (*F*[3, 39] = 14.64, *P* < 0.001, *η^2^_p_* = 0.53). There were significant increases in $\dot{V}_{{CO}_{2}}$in both conditions, and $\dot{V}_{{CO}_{2}}$during exercise in HE conditions was higher compared with ME conditions (*t*[13] = 3.104, *P* < 0.01, *d* = 0.25, paired *t*-test). ETO_2_ significantly increased during exercise in ME conditions (*t*[13] = -7.34, *P* < 0.001, *d* = 2.56, paired *t*-test) compared with HE conditions and decreased during post-Stroop sessions in HE conditions compared to pre-Stroop sessions (*t*[13] = 6.520, *P* < 0.01, *d* = 0.89, paired *t*-test).

There was no notable correlation between the RT and any parameter. Moreover, we examined the correlation between reduced activation in the l-DLPFC and altered respiratory parameters during exercise under the HE condition. There was no significant correlation between l-DLPFC activation and any of the parameters.
